# Hyperparathyroidism: current concepts in diagnosis and surgical treatment

**DOI:** 10.3389/fendo.2026.1809252

**Published:** 2026-06-26

**Authors:** Zhiyuan Wang, Xianqiang Yu

**Affiliations:** Department of Thyroid Disease Diagnosis and Treatment, Qingdao Municipal Hospital (Qingdao Hospital of Rehabilitation University), Qingdao, China

**Keywords:** hungry bone syndrome (HBS), hyperparathyroidism (HPT), hypocalcemia, parathyroid hormone (PTH), parathyroidectomy

## Abstract

Hyperparathyroidism (HPT) is a complex endocrine disorder characterized by the dysregulated secretion of parathyroid hormone (PTH), leading to significant disruptions in calcium and phosphate homeostasis. This review provides a comprehensive analysis of the condition, beginning with the distinct clinical and biochemical profiles of its primary, secondary, and tertiary forms. It critically examines the evolving diagnostic pathways and the refined indications for surgical intervention, which remains the only curative treatment for primary HPT and a crucial therapy for advanced secondary and tertiary disease. Central to the discussion is a detailed exploration of modern surgical strategies, highlighting the paradigm shift from traditional bilateral neck exploration to precision-focused, minimally invasive parathyroidectomy, enabled by advanced preoperative localization and intraoperative PTH monitoring. The review further considers outcomes, postoperative management, and emerging techniques, including remote-access endoscopic approaches. By synthesizing current evidence and guidelines, this work aims to delineate a holistic framework for the effective management of HPT, from accurate diagnosis through to the selection and execution of the optimal surgical treatment strategy.

## 1 Introduction

Hyperparathyroidism (HPT) is a complex endocrine disorder characterized by the inappropriate and excessive secretion of parathyroid hormone (PTH), which disrupts the critical balance of calcium and phosphate metabolism (1–3). This dysregulation manifests as a clinical spectrum, traditionally classified into three distinct etiological categories: primary HPT (PHPT), resulting from autonomous overactivity of one or more parathyroid glands; secondary HPT (SHPT), a physiological compensatory response to chronic hypocalcemic states such as chronic kidney disease or vitamin D deficiency; and tertiary HPT (THPT), in which prolonged secondary stimulation leads to autonomous glandular function (4–7). Primary hyperparathyroidism is characterized by autonomous overproduction of PTH from one or more parathyroid glands, most commonly due to a solitary parathyroid adenoma (80-85%), parathyroid hyperplasia (10-15%), or, rarely, parathyroid carcinoma. The excess PTH drives hypercalcemia by increasing bone resorption, enhancing intestinal calcium absorption via calcitriol activation, and promoting renal tubular calcium reabsorption. Secondary hyperparathyroidism represents a compensatory increase in PTH secretion in response to chronic hypocalcemia or hyperphosphatemia, most commonly resulting from chronic kidney disease, vitamin D deficiency, or malabsorption syndromes. Unlike primary hyperparathyroidism, serum calcium is typically low or normal, while PTH is elevated. While classic symptomatology is well-documented, the modern epidemiological profile reveals that a majority of patients, particularly in Western populations, are diagnosed incidentally through routine biochemical screening, presenting with subtle or absent symptoms (8, 9). If the underlying cause is not corrected, prolonged secondary hyperparathyroidism can lead to skeletal complications including renal osteodystrophy, and in some cases, may progress to tertiary hyperparathyroidism, characterized by autonomous PTH secretion even after correction of the initial stimulus. In patients with end-stage renal disease awaiting kidney transplantation, surgical parathyroidectomy is often required when medical management fails to control severe hyperparathyroidism, particularly when preoperative PTH levels remain markedly elevated despite optimal therapy, as persistent hyperparathyroidism post-transplantation can compromise allograft function and increase the risk of fracture and cardiovascular complications. This shift underscores the necessity for nuanced diagnostic and therapeutic frameworks.

The definitive management of HPT, surgical intervention remains the definitive treatment for HPT, is surgical intervention. Parathyroidectomy stands as the only curative treatment, capable of normalizing biochemical parameters, resolving debilitating symptoms, and halting progressive end-organ damage to the skeletal and renal systems (10–14). The surgical approach to HPT has undergone a profound transformation over the past quarter-century, driven by advancements in preoperative localization and intraoperative decision-making. The advent of high-sensitivity imaging, such as technetium-99m sestamibi scintigraphy and high-resolution ultrasonography, combined with the widespread adoption of rapid intraoperative PTH monitoring, has facilitated a paradigm shift from routine bilateral neck exploration to targeted, minimally invasive parathyroidectomy (15–18). This evolution has resulted in superior patient outcomes, achieving cure rates exceeding 95% while significantly reducing operative morbidity, hospital stay, and recovery time. In recent years, fluorocholine PET/CT has emerged as a pivotal imaging modality for preoperative localization of hyperfunctioning parathyroid glands, particularly when conventional imaging modalities such as ultrasonography and technetium-99m sestamibi scintigraphy are negative or discordant. Fluorocholine PET/CT exploits the increased choline metabolism in hyperfunctioning parathyroid tissue, demonstrating superior sensitivity for detecting ectopic glands, multiglandular disease, and mediastinal or intrathyroidal adenomas that may be missed by other techniques. This advanced imaging has proven especially valuable in the setting of persistent or recurrent hyperparathyroidism after prior surgery, where distorted anatomy and fibrosis complicate reoperative exploration, and in patients with multiglandular disease, where accurate preoperative localization can guide the extent of surgical resection and reduce the risk of postoperative hypoparathyroidism or persistent disease.

This academic review aims to provide a comprehensive and critical synthesis of the contemporary understanding of hyperparathyroidism, with a principal focus on the evolution and current state of its surgical management. We will first delineate the pathophysiological characteristics, diagnostic criteria, and clinical manifestations of the primary, secondary, and tertiary forms of HPT. Subsequently, the discussion will concentrate on the surgical landscape, meticulously examining preoperative assessment strategies, refined operative indications, the spectrum of surgical techniques, and essential postoperative management. By contextualizing these elements within the latest clinical guidelines and evidence-based practices, this review seeks to elucidate the pivotal role of surgery in the multidisciplinary care pathway for HPT, serving as a consolidated resource for clinicians and surgeons dedicated to optimizing patient outcomes in this complex endocrine disease.

## 2 Clinical manifestations and diagnostic approach

The clinical presentation of hyperparathyroidism (HPT) spans a broad and often non-specific spectrum, traditionally encapsulated by the mnemonic “stones, bones, groans, and psychic overtones,” reflecting its multi-system impact (19, 20). Classic symptomatic primary HPT (PHPT) manifests through renal complications such as nephrolithiasis and nephrocalcinosis; skeletal disorders including osteitis fibrosa cystica, osteoporosis, and bone pain; gastrointestinal disturbances like peptic ulcer disease and pancreatitis; and neuropsychiatric symptoms of fatigue, depression, and cognitive dysfunction (21–23). However, the epidemiological landscape in developed nations has shifted dramatically, with over 80% of contemporary PHPT cases diagnosed incidentally through routine laboratory testing, presenting as “asymptomatic” or with subtle, vague complaints. This silent phenotype, coupled with the distinct clinical pictures of secondary HPT (SHPT) driven by chronic kidney disease and marked by renal osteodystrophy, vascular calcification, and pruritus and tertiary HPT (THPT), necessitates a meticulous and systematic diagnostic approach to distinguish HPT from other causes of calcium dysregulation (24–27). THPT is a condition of autonomous parathyroid hormone (PTH) overproduction that typically develops in patients with long-standing SHPT, most commonly those with chronic kidney disease or after kidney transplantation. This transition from “secondary” to “tertiary” occurs because prolonged stimulation causes the parathyroid cells to become autonomous.

Definitive diagnosis hinges on a consistent biochemical profile, beginning with the confirmation of persistent hypercalcemia on at least two separate measurements. The cornerstone of diagnosing PHPT is the concurrent finding of an inappropriately elevated or non-suppressed serum parathyroid hormone (PTH) level in the setting of hypercalcemia (12, 28). A comprehensive panel should also include serum phosphate, creatinine, and 25-hydroxyvitamin D (29–31). Crucially, the differential diagnosis must exclude familial hypocalciuric hypercalcemia (FHH), a benign condition mimicking PHPT. This is achieved by calcium/creatinine clearance ratio (CCCR), which is typically low in FHH but normal or elevated in PHPT. For SHPT, the biochemical signature is characterized by elevated PTH levels in the context of normal or low serum calcium, usually secondary to chronic hypocalcemic triggers.

Following biochemical confirmation, the diagnostic pathway diverges into localization and assessment of end-organ damage. Preoperative localization for surgical planning in PHPT primarily employs high-resolution cervical ultrasound and technetium-99m sestamibi scintigraphy, often with single-photon emission computed tomography (SPECT/CT) (15–17). These imaging modalities aim to identify the culprit adenoma and are particularly vital for planning minimally invasive surgery. This structured diagnostic cascade from identifying biochemical abnormalities to localizing anatomical lesions and quantifying clinical impact is essential for accurate diagnosis, risk stratification, and guiding appropriate therapeutic intervention.

## 3 Surgical management: indications and operative strategies

The decision to pursue parathyroidectomy is guided by a synthesis of robust clinical guidelines and patient-centered evaluation. For primary hyperparathyroidism (PHPT), surgical intervention is unequivocally recommended for all symptomatic patients addressing manifestations such as nephrolithiasis, overt bone disease, and neurocognitive dysfunction (32–34). In the prevalent asymptomatic cohort, operative criteria are defined by objective parameters including a serum calcium elevation >1.0 mg/dL above the normal range, a glomerular filtration rate reduced below 60 mL/min, a bone mineral density T-score of -2.5 or lower, the occurrence of a vertebral fragility fracture, or age younger than 50 years (5, 35–38). For secondary and tertiary HPT, surgery becomes a critical intervention when medical management fails to control severe hypercalcemia, progressive skeletal pain, calciphylaxis, or significant glandular enlargement.

Normocalcemic primary hyperparathyroidism (NPHPT) is a distinct variant of primary hyperparathyroidism characterized by persistently elevated parathyroid hormone (PTH) levels in the setting of consistently normal serum calcium concentrations, after excluding secondary causes of hyperparathyroidism such as vitamin D deficiency, chronic kidney disease, or medications (5, 33). Patients with NPHPT represent a clinically heterogeneous group. Some remain asymptomatic with stable biochemical profiles over years, while others progress to hypercalcemic primary hyperparathyroidism or develop complications including reduced bone mineral density, increased fracture risk, nephrolithiasis, and neurocognitive symptoms, though the natural history remains less well-defined than in classic hypercalcemic disease. The workup of suspected NPHPT requires careful exclusion of secondary causes. Initial evaluation should include measurement of serum calcium, intact PTH, 25-hydroxyvitamin D, creatinine with estimated glomerular filtration rate, and 24-hour urinary calcium excretion. Vitamin D deficiency must be corrected before confirming the diagnosis, as low vitamin D can appropriately stimulate PTH secretion (35). Additional assessments include bone mineral density by dual-energy X-ray absorptiometry at the lumbar spine, hip, and distal radius, renal imaging to screen for nephrolithiasis or nephrocalcinosis, and evaluation for potential end-organ effects. Surgical management of NPHPT remains controversial, as clear consensus guidelines are less established than for classic hypercalcemic disease. Parathyroidectomy is generally recommended for patients with NPHPT who demonstrate end-organ manifestations, including osteoporosis, nephrolithiasis, or progressive disease. Surgery may also be considered in younger patients or those with symptoms potentially attributable to hyperparathyroidism after excluding alternative etiologies.

The execution of surgical cure has evolved into a strategic algorithm, balancing disease characteristics with procedural sophistication. Traditional bilateral neck exploration (BNE) remains the foundational and indispensable open surgical approach (39–43). Performed through a transverse cervical incision, BNE provides direct panoramic visualization of the central neck compartment enabling the systematic identification of all parathyroid glands. This comprehensiveness establishes BNE as the gold standard for cases of multiglandular hyperplasia, negative or discordant preoperative localization, familial HPT syndromes, and reoperative surgery. Its enduring role is to ensure anatomical and biochemical success in the most complex presentations, where the surgeon’s ability to assess all glands intraoperatively is paramount to achieving a durable cure.

In contrast, for the majority of sporadic PHPT caused by a single, pre-localized adenoma, minimally invasive parathyroidectomy (MIP) represents the contemporary standard of care (44–48). This paradigm is predicated on the synergy of precise preoperative imaging and the critical use of intraoperative parathyroid hormone (IOPTH) monitoring (49, 50). The focused, gland-directed nature of MIP allows for a significant reduction in surgical footprint, most commonly through a small, targeted incision directly over the adenoma. The biochemical confirmation provided by a >50% drop in IOPTH levels at 10 minutes post-resection reliably predicts operative success, permitting the safe conclusion of surgery without routine visualization of all glands, thereby minimizing dissection, postoperative discomfort, and recovery time.

The selection between these two principal strategies is a nuanced clinical judgment. The minimally invasive approach is favored for its targeted efficiency, reduced morbidity, and superior cosmetic outcome in appropriate candidates with concordant imaging. However, the traditional open approach retains its critical importance as a comprehensive and adaptable strategy for complex disease, where anatomical certainty supersedes the benefits of minimal access. Ultimately, the surgical management of HPT is characterized by this duality of purpose: the application of precise, targeted therapy for straightforward disease, and the availability of definitive, exploratory intervention for complex cases. The surgeon’s expertise in preoperative evaluation, mastery of both techniques, and judicious use of IOPTH monitoring are the cornerstones of navigating this spectrum to achieve optimal patient outcomes.

IOPTH monitoring is a critical adjunct during parathyroidectomy, serving as a real-time biochemical confirmation of complete resection and playing an essential role in excluding unsuspected multiglandular disease. The standard IOPTH protocol involves obtaining a baseline PTH level after induction of anesthesia but before skin incision, followed by measurements at 5 and 10 minutes after excision of the suspected hyperfunctioning gland(s). A decrease of ≥50% from the baseline level at 10 minutes post-excision, with the absolute PTH value ideally falling into the normal range, reliably confirms that all hypersecreting tissue has been removed (51–53). Conversely, failure to achieve this adequate decrease strongly suggests the presence of additional hyperfunctioning glands prompting the surgeon to extend exploration rather than terminating the procedure prematurely. This capability is particularly valuable in patients with multiglandular hyperplasia or in normocalcemic primary hyperparathyroidism where multiglandular involvement occurs more frequently than in classic hypercalcemic disease, as IOPTH allows dynamic assessment of whether resection of a single macroscopically enlarged gland has sufficiently corrected the biochemical abnormality or whether further exploration is required. By guiding the extent of resection, IOPTH helps reduce the risk of persistent or recurrent hyperparathyroidism from missed multiglandular disease while also preventing unnecessary extensive bilateral neck exploration when single-gland disease is confirmed, thereby optimizing surgical outcomes.

The evolution of parathyroid surgery for primary hyperparathyroidism (PHPT) has increasingly favored minimally invasive approaches over traditional bilateral neck exploration, with focused parathyroidectomy, endoscopic parathyroidectomy, and radio-guided parathyroidectomy representing three key strategies, each offering distinct advantages particularly regarding cosmetic outcomes (54). Focused parathyroidectomy, performed through a small (2–3 cm) unilateral incision, has become the most widely adopted minimally invasive technique due to its shorter operative time and favorable cure rates exceeding 95% (55). A prospective randomized trial comparing focused versus conventional bilateral exploration found that focused parathyroidectomy resulted in significantly lower postoperative pain intensity at multiple time points (p < 0.001), reduced analgesic consumption (p < 0.001), shorter scar length (p < 0.001), and higher cosmetic satisfaction rates at 2 days, 1 month, and 6 months after surgery (p < 0.001 for early time points) (55). However, when cosmetic considerations are paramount, endoscopic parathyroidectomy offers the advantage of leaving no visible neck scar, as the incisions are concealed inside the oral vestibule (56). A randomized clinical trial by Meshkati Yazd et al. directly compared endoscopic versus focused parathyroidectomy and found that while focused surgery had significantly shorter operative time (p < 0.001), the endoscopic group achieved significantly higher patient satisfaction with cosmetic outcomes and a faster biochemical success rate at five minutes (95.3% vs. 77.1%, p = 0.013) (57). The largest TOEPVA series to date reported a 95.2% success rate, with the only conversion occurring for a retro-esophageal adenoma (56). The lateral endoscopic approach, first described by Henry et al. provides excellent access to posteriorly located adenomas through the plane between the strap muscles and carotid sheath, with a prospective study of 200 patients reporting a 98% cure rate (54). Meanwhile, radio-guided parathyroidectomy, which utilizes preoperative injection of 99mTc-sestamibi and intraoperative gamma-probe detection, enables precise adenoma localization even in challenging scenarios such as ectopic glands or reoperative necks (58). This finding challenges the assumption that simply miniaturizing neck incisions adequately addresses cosmetic concerns and supports the development of remote-access techniques. Collectively, these minimally invasive options have shifted the paradigm from “cure alone” to “cure with optimal cosmetic and functional outcomes”.

While both secondary hyperparathyroidism (SHPT) and tertiary hyperparathyroidism (THPT) arise in the context of chronic kidney disease (CKD), their underlying pathophysiology and surgical indications differ fundamentally. SHPT is a compensatory response to persistent hypocalcemia, hyperphosphatemia, and vitamin D deficiency in patients with CKD, and operative intervention is generally indicated when medical management fails to control severe biochemical abnormalities, progressive hypercalcemia or hyperphosphatemia despite optimal therapy, or when complications such as calciphylaxis, severe bone pain, fractures, or extensive vascular calcifications develop. In contrast, THPT represents autonomous parathyroid overactivity that persists or develops after restoration of normal renal function, typically following kidney transplantation. According to Dulfer et al., operative intervention for THPT is indicated when autonomous PTH secretion persists for more than one year after a successful kidney transplant in patients who also demonstrate additional clinical or biochemical features, including hypophosphatemia, low bone mineral density or severe osteopenia, fatigue, pruritus, bone pain, peptic ulcer disease, or nephrocalcinosis (59). The rationale for this one-year observation period is that some patients experience spontaneous resolution of hyperparathyroidism within the first-year post-transplant as the skeletal resistance to PTH improves and calcium homeostasis normalizes.

Tertiary hyperparathyroidism commonly presents significant diagnostic overlap with primary hyperparathyroidism due to nearly identical core biochemical profiles, including persistent hypercalcemia and inappropriately elevated or unsuppressed parathyroid hormone levels, which precludes reliable differentiation based solely on laboratory parameters. However, the two entities can be reliably distinguished by their distinct clinical contexts and pathogenetic backgrounds. Primary hyperparathyroidism arises from autonomous, unregulated parathyroid gland overactivity driven by intrinsic parathyroid lesions such as adenomas, hyperplasia, or carcinoma, occurring in patients with essentially normal renal function and no history of chronic secondary parathyroid stimulation. In contrast, tertiary hyperparathyroidism evolves as a late complication of long-standing secondary hyperparathyroidism, most frequently caused by chronic kidney disease, end-stage renal disease, or prolonged malabsorption-related hypocalcemia and hypovitaminosis D, wherein chronic physiological stimulation gradually induces irreversible, autonomous parathyroid hyperfunction and parathyroid gland hyperplasia even after the resolution of the original hypocalcemic stimulus. Therefore, while biochemical testing alone fails to differentiate these two conditions, the presence of a prolonged clinical history of chronic kidney disease, long-term secondary hyperparathyroidism, or prior renal transplantation serves as the definitive clinical clue to establish the diagnosis of tertiary hyperparathyroidism, guiding clinicians to distinguish it from *de novo* primary hyperparathyroidism and avoid misdiagnosis.

Persistent disease beyond this time point, however, is unlikely to resolve spontaneously and warrants surgical consideration to prevent long-term complications such as progressive bone loss, nephrolithiasis, and allograft dysfunction. Therefore, while SHPT surgery is often driven by refractory biochemical abnormalities and end-organ damage, THPT surgery is more precisely guided by the combination of prolonged post-transplant autonomy (≥1 year) and specific symptomatic or metabolic derangements.

In addition to definitive surgical intervention, contemporary management of hyperparathyroidism encompasses standardized conservative and medical treatment strategies for patients who are ineligible for parathyroidectomy or decline surgical resection, a clinically important cohort that predominately includes elderly individuals, multimorbid patients with severe underlying systemic diseases, and those with mild, asymptomatic hyperparathyroidism not meeting surgical criteria per current clinical guidelines. Pharmacological therapy in this setting primarily focuses on skeletal protection, bone mineral density preservation and improvement, and normalization of abnormal calcium and parathyroid hormone levels to mitigate long-term skeletal and cardiovascular complications. Beyond skeletal protection, calcimimetics are recommended in clinical practice to suppress excessive parathyroid hormone secretion and correct hypercalcemia, particularly beneficial for patients with persistent biochemical abnormalities and advanced disease. Collectively, these guideline-consistent pharmacological approaches constitute a comprehensive conservative management framework, optimizing clinical outcomes and reducing disease-related morbidity in the growing population of patients managed with non-surgical strategies in real-world clinical practice.

## 4 Postoperative management and outcomes

Immediate postoperative management centers on vigilant monitoring for two primary complications: hypocalcemia and hematoma formation (32, 60–62). Transient hypocalcemia is common, resulting from suppressed function of the remaining normal glands or, in cases of severe bone disease, from a dramatic influx of calcium into remineralizing bones. Patients are monitored for symptoms of paresthesia, muscle cramping, or Chvostek’s and Trousseau’s signs (63). Symptomatic or profound hypocalcemia is managed with intravenous calcium gluconate, followed by a transition to high-dose oral calcium carbonate and active vitamin D. Serum calcium and PTH levels are typically measured within 6–24 hours postoperatively to confirm early biochemical normalization, while cure is usually defined at 6 months. While rare, a rapidly expanding cervical hematoma constitutes a surgical emergency requiring immediate bedside decompression and operative evacuation.

The outcomes of parathyroidectomy are overwhelmingly favorable, establishing it as one of the most successful and curative procedures in endocrine surgery. For primary hyperparathyroidism (PHPT), cure rates, defined by normocalcemia lasting at least six months, exceed 95% in initial operations at high-volume centers (1, 64–66). Successful surgery leads to rapid normalization of serum calcium and PTH, 8-12% bone mineral density increase in lumbar spine, and a significant reduction in the risk of new kidney stone formation. Symptomatic patients often report dramatic improvements in fatigue, bone pain, and neurocognitive complaints. Complication rates are low, with permanent recurrent laryngeal nerve injury and permanent hypoparathyroidism each occurring in less than 1-2% of cases when performed by experienced surgeons (67–69). These excellent outcomes underscore parathyroidectomy as a highly effective intervention with a profound positive impact on patient quality of life and long-term health.

A distinct and clinically critical postoperative complication following parathyroidectomy is hungry bone syndrome (HBS), a transient but potentially severe metabolic disorder characterized by rapid and robust skeletal remineralization after the abrupt correction of chronic hyperparathyroidism (70, 71). The underlying pathophysiology stems from long-standing excessive parathyroid hormone-driven osteoclastic bone resorption, which induces profound demineralization and increased skeletal turnover. Following successful parathyroid resection, the sudden withdrawal of hyperparathyroid stimulus shifts bone metabolism toward dominant osteoblastic activity, resulting in avid systemic uptake of circulating minerals for rapid bone reconstruction. Biochemically, HBS extends beyond isolated postoperative hypocalcemia, and consistently presents with concurrent hypophosphatemia due to accelerated skeletal phosphate deposition, alongside variable hypomagnesemia and suppressed parathyroid hormone levels during the early postoperative period. Several well-defined preoperative risk factors predispose patients to HBS, including severe and long-standing hyperparathyroidism, markedly elevated serum alkaline phosphatase reflecting high bone turnover, extensive preoperative skeletal disease and osteoporotic changes, concomitant vitamin D deficiency, and the presence of large parathyroid adenomas or multiglandular disease with substantial parathyroid hormone elevation (72, 73). Recognition of these preoperative risk factors and the full biochemical spectrum of HBS enables early risk stratification, targeted postoperative monitoring, and timely calcium and vitamin D supplementation, which are essential to prevent symptomatic hypocalcemia and optimize perioperative patient management.

## 5 Discussion and future directions

The evolution in the management of hyperparathyroidism from mandatory open exploration to image-guided, minimally invasive, and remote-access surgery represents a triumph of precision medicine in endocrine surgery. The integration of high-resolution preoperative imaging and rapid intraoperative PTH monitoring has successfully decoupled surgical success from the necessity of visualizing all glands, thereby reducing patient morbidity without compromising cure rates. However, this paradigm also presents ongoing challenges. Accurate identification of multiglandular disease preoperatively remains imperfect, and the management of normocalcemic primary hyperparathyroidism continues to spark debate regarding its natural history and optimal intervention timing. Furthermore, while remote-access techniques offer superior cosmesis, their broader adoption is tempered by longer operative times, steep learning curves, and procedure-specific complication profiles that necessitate rigorous cost-benefit analysis and surgeon training. The discussion also extends to secondary hyperparathyroidism, where the role of surgery is being continually redefined against advances in pharmacotherapy with calcimimetics, demanding clearer guidelines on the timing of surgical referral.

Future directions in the field are poised to leverage technological and biological innovations to further personalize care. Enhanced molecular profiling of parathyroid tissue, including genetic and epigenetic markers, may predict tumor behavior, identify hereditary syndromes earlier, and clarify the pathogenesis of atypical adenomas and carcinoma. In diagnostics, artificial intelligence (AI) applied to preoperative imaging and clinical data holds promise for improving localization accuracy and predicting multiglandular involvement. AI-assisted analysis of imaging modalities such as ultrasound and SPECT/CT may improve localization accuracy and help predict multiglandular disease. Therapeutically, for patients who are poor surgical candidates, image-guided thermal ablation techniques are under active investigation as potential non-surgical alternatives for localized disease, though long-term efficacy data are awaited. Concurrently, the refinement of existing minimal-access approaches and the development of next-generation robotic platforms aim to improve ergonomics and outcomes in remote-access surgery. Ultimately, the trajectory points toward a future where treatment algorithms are increasingly tailored, integrating genomic data, advanced imaging analytics, and a wider array of therapeutic modalities to optimize outcomes for every patient across the spectrum of hyperparathyroid disease.

## 6 Conclusion

In summary, the management of hyperparathyroidism (HPT) exemplifies the successful convergence of precise diagnostic stratification and continually refined surgical technique. From the foundational bilateral neck exploration to the contemporary growing use of minimally invasive and, in select centers, remote-access endoscopic approaches, surgical intervention remains the definitive and curative standard, particularly for primary HPT, achieving biochemical cure and symptom resolution in over 95% of cases. The critical synergy of accurate preoperative localization, the decisive guidance of intraoperative parathyroid hormone monitoring, and adherence to clear, evidence-based operative indications forms the cornerstone of optimal patient outcomes. As the field progresses, the integration of molecular diagnostics, artificial intelligence, and novel ablative technologies promises to further personalize therapeutic pathways, ensuring that the surgical treatment of hyperparathyroidism continues to advance in efficacy, precision, and alignment with individual patient needs and preferences.
